# Human endogenous retroviruses and cancer prevention: evidence and prospects

**DOI:** 10.1186/1471-2407-13-4

**Published:** 2013-01-03

**Authors:** Luca Cegolon, Cristiano Salata, Elisabete Weiderpass, Paolo Vineis, Giorgio Palù, Giuseppe Mastrangelo

**Affiliations:** 1 Department of Molecular Medicine, Padua University, Padua, Italy; 2 Imperial College London, School of Public Health, S. Mary’s Campus, London, UK; 3 Department of Community Medicine, Faculty of Health Sciences, University of Tromsø, Tromsø, Norway; 4 Department of Research, Cancer Registry of Norway, Oslo, Norway; 5 Department of Medical Epidemiology and Biostatistics, Karolisnska Institute, Stockholm, Sweden; 6 Samfundet Folkhälsan, Helsinki, Finland; 7 HuGeF Foundation, Turin, Italy

**Keywords:** HERV-K, Cancer prevention, Melanoma, Breast cancer, Ovarian cancer, BCG, Vaccinia, Yellow fever virus vaccine, Epidemiology

## Abstract

**Background:**

Cancer is a significant and growing problem worldwide. While this increase may, in part, be attributed to increasing longevity, improved case notifications and risk-enhancing lifestyle (such as smoking, diet and obesity), hygiene-related factors resulting in immuno-regulatory failure may also play a major role and call for a revision of vaccination strategies to protect against a range of cancers in addition to infections.

**Discussion:**

Human endogenous retroviruses (HERVs) are a significant component of a wider family of retroelements that constitutes part of the human genome. They were originated by the integration of exogenous retroviruses into the human genome millions of years ago. HERVs are estimated to comprise about 8% of human DNA and are ubiquitous in somatic and germinal tissues.

Physiologic and pathologic processes are influenced by some biologically active HERV families. HERV antigens are only expressed at low levels by the host, but in circumstances of inappropriate control their genes may initiate or maintain pathological processes. Although the precise mechanism leading to abnormal HERVs gene expression has yet to be clearly elucidated, environmental factors seem to be involved by influencing the human immune system.

HERV-K expression has been detected in different types of tumors.

Among the various human endogenous retroviral families, the K series was the latest acquired by the human species. Probably because of its relatively recent origin, the HERV-K is the most complete and biologically active family.

The abnormal expression of HERV-K seemingly triggers pathological processes leading to melanoma onset, but also contributes to the morphological and functional cellular modifications implicated in melanoma maintenance and progression.

The HERV-K-MEL antigen is encoded by a pseudo-gene incorporated in the HERV-K *env*-gene. HERV-K-MEL is significantly expressed in the majority of dysplastic and normal naevi, as well as other tumors like sarcoma, lymphoma, bladder and breast cancer. An amino acid sequence similar to HERV-K-MEL, recognized to cause a significant protective effect against melanoma, is shared by the antigenic determinants expressed by some vaccines such as BCG, vaccinia virus and the yellow fever virus.

HERV-K are also reactivated in the majority of human breast cancers. Monoclonal and single-chain antibodies against the HERV-K Env protein recently proved capable of blocking the proliferation of human breast cancer cells in vitro, inhibiting tumor growth in mice bearing xenograft tumors.

**Summary:**

A recent epidemiological study provided provisional evidence of how melanoma risk could possibly be reduced if the yellow fever virus vaccine (YFV) were received at least 10 years before, possibly preventing tumor initiation rather than culling melanoma cells already compromised. Further research is recommended to confirm the temporal pattern of this protection and eliminate/attenuate the potential role of relevant confounders as socio-economic status and other vaccinations.

It appears also appropriate to examine the potential protective effect of YFV against other malignancies expressing high levels of HERV-K antigens, namely breast cancer, sarcoma, lymphoma and bladder cancer.

Tumor immune-therapy, as described for the monoclonal antibodies against breast cancer, is indeed considered more complex and less advantageous than immune-prevention. Cellular immunity possibly triggered by vaccines as for YFV might also be involved in anti-cancer response, in addition to humoral immunity.

## Background

Cancer is a significant and growing problem worldwide [1,2]. In the United Kingdom, for example, 42% of people who died in 2008 had a diagnosis of cancer sometime in their life, and tumors were the cause of death in 64% of these patients [3].

The improvement of survival observed in the past 20 years is associated with a marked increase in the average treatment cost for most common cancers [4,5]. The new targeted cancer treatments are expected to raise even more abruptly in the next future [6], especially in developed countries such the US where the population older than 65 is expected to almost double in 2030 [7].

Although improved case notifications, increasing longevity and risk-enhancing lifestyle (such as smoking, diet and obesity) have to be taken into account, the burden of cancer may in part be attributed also to hygiene-related factors resulting in immuno-regulatory failure [8,9]. The latter call for a revision of vaccination strategies to protect against a range of cancers in addition to infections.

## Discussion

### Human endogenous retroviruses

The human genome contains around 400,000 genetic loci [10], evolved as a result of past infection by many different kinds of retroviruses. Approximately 45% of human genome is actually composed of or derived from virus-like transposon-related elements [11,12].

Germ cell infections by exogenous retro-viruses occurred millions of years ago and led to the stable maintenance of human endogenous retroviruses (HERVs) into the human genome. The integration of HERVs into the host cell happens within the context of their replication cycle [13,14]. HERVs are estimated to comprise about 8% of human DNA [15,16] and two hypotheses have been suggested to justify their persistence in the human genome during evolution. According to the parasitic theory HERVs were neutral and their elimination was rather difficult [17-21]. Conversely, the symbiotic theory sees them retained by positive selection, provided their function was relevant to maintain certain vital conditions [22]. However, the two hypotheses are not mutually exclusive, as after the initial integration, subsequent random mutations of the parasitic viral RNA of HERVs led to the synthesis of important human proteins, enabling retroviruses to persist in the human DNA as symbiotic. Zeyl [23] recently reviewed the significance of symbiotic DNA in eukaryotes.

Unlike typical viruses, HERVs are not infectious [15,24], but they can be transmitted vertically as pro-viruses in a Mendelian fashion [25]; furthermore as a consequence of multiple mutations and deletions, they are defective and therefore unable to retro-transpose [26].

### HERV expression

After integrating into the host DNA, HERVs can produce hundreds of copies of themselves and newly integrate throughout the human genome. HERV genes *gag, pol* and *env* are flanked by genetic regulatory sequences named Long Terminal Repeats (LTRs), used by HERV to insert their genetic sequences into the host DNA and able to regulate both retroviral and sometimes functional human genes.

HERVs generally become non replication competent by recombinational deletion between the two LTRs and/or by random mutations occurring while the host genome is undergoing DNA replication. However, complete or incomplete gene products can be either directly coded by HERV genes e*nv* or *gag* or result from recombinational mechanisms [27]. Physiologic and pathologic processes are influenced by some biologically active HERV families through direct RNA viral transcripts or mutations generated by retro-transposition [28]. As mentioned earlier HERVs indeed code for fundamental human proteins and have been highly involved in the intra-uterine development of the fetus as well as in the evolution of the human species [29,30]. The *env* region of three HERVs (ERV-3, HERV-W and HERV-FRD) is crucial to form the placental syncytiotrophoblast, and HERV-FRD seems also to contribute in down-regulation of human immunity against the fetus and prevent its rejection [31,32].

HERV antigens are only expressed at low levels by the host, but in circumstances of inappropriate control the expression of HERV genes may initiate or maintain pathological processes [33]. According to microarray analysis, HERV expression appears to be positively influenced by the exposure to exogenous (e.g chemicals, UV radiations [34,35]) and endogenous (e.g. cytokines, hormones [34,36,37]) stimuli.

Although the precise mechanism leading to abnormal HERVs gene expression has to be further elucidated, environmental factors seem to be involved by influencing the human immune system [38], and hypo-methylation of the relevant retroviral genes appears a key factor [39,40].

### The HERV-K family

HERVs are classified in more than 22 different families [15,41-44] depending on their sequence identity and partly on the similarity of their primer binding sites to host tRNAs [15,44,45].

Among the various human endogenous retroviral families, the K series was the latest acquired by the human species, between three and six million years ago [46]. Probably because of this relatively recent origin, the HERV-K is the most complete and biologically active family, being composed of retro-elements showing polymorphic integration in the human genome [15,43,47,48].

HERV-K is the only known retroviral family that has retained functional full-length open reading frames (ORF) coding for structural and enzymatic proteins [15,49,50] and appears capable to induce the generation of replicating viral components [29,47,51].

HERV-K encoding loci are thought to be transcriptionally silent in normal cells, becoming active after malignant transformation, as found in germ cell tumors [52]. Activation of HERV-K may initiate or maintain carcinogenesis.

HERV-K expression was detected in different types of tumors and Hill’s causal criteria for epidemiology have been recently adapted to assess virus-cancer associations [53]):

*consistency of association*. Transcripts of HERVs have been detected by many independent investigators in different tumors: breast cancer [25,54-60], ovarian cancer [61], lymphoma [54], melanoma [25,62,63], germ line tumors [51,60,64], haematological neoplasms [65,66], sarcoma [25], bladder and prostate cancer [25], primary skin tumours and lymphatic metastases [50,55];

*strength of the association*. HERV genes are rarely expressed in normal tissues [25,67] and adjacent tissues of breast [58] and other types of cancers [68];

*temporality*. Environmental factors − both exogenous (chemicals [35], UV radiation, [34,69,70], smoking [71], viruses [72]) and endogenous (estrogen [36], and cytokines [37]) − facilitate HERV expression;

*biological plausibility*. HERV proteins reduce expression of glutathione peroxidase, thus increasing the levels of reactive oxygen species with subsequent cumulative cell damage [73];

*experimental evidence*. Vaccinating against a peptide from a mouse endogenous retrovirus was shown to prevent, though not to cure, established melanoma in mice [74].

### HERV-K and Melanoma

**Figure 1:**
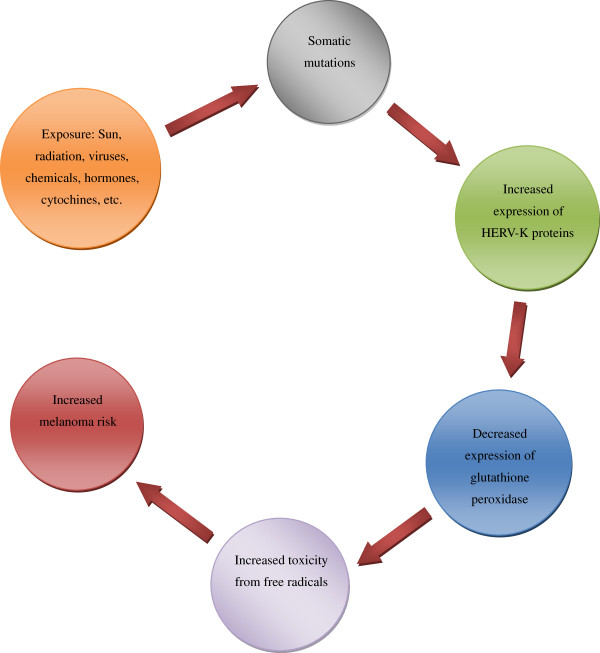
**Cascade of events due to homology sequence between the HERV-K Env protein and OREBP**[38].

The abnormal expression of HERV-K seemingly triggers the pathological processes leading to melanoma onset, but also contributes to the morphological and functional cellular modifications implicated in melanoma maintenance and progression [62]. Figure 1 shows the presumed cascade of events between HERV-K expression and melanoma initiation. The molecular mimicry of HERV-K transcript with Oxygen Responsive Element Binding Protein (OREBP) decreases the expression of glutathione peroxidase and increases the toxicity from free radicals leading to higher risk of cancer [38].

Conversely to benign melanocytic lesions, specimen from patients with primary or metastatic melanoma as well as melanoma biopsy-derived cell lines were reported to express HERV-K antigens such as the viral reverse transcriptase (RT) [50,55]. Down-regulation (by RNA interference) and pharmacological inhibition of RT resulted in a reduced proliferation, induced morphological differentiation and reprogrammed gene expression in melanoma cells. Discontinuation of anti-RT treatment reversed the latter figures, suggesting a possible epigenetic level of control by RT [75].

Down-regulation of HERV-K led to rejection of melanoma cells in immune-competent mice [76] and decreased cancerogenic capacity of melanoma cells inoculated into nude mice [77]. It has been hypothesized that HERV-K expression contributes to evade immune-surveillance in immune-competent mice, thus promoting the growth of transformed cells and stimulating tumour progression [63,77].

An immune-dominant epitope on the *Env* protein that is recognized by antibodies from sera of patients with melanoma. The prevalence of antibodies against the immune-dominant epitope of the HERV-K Env protein was significantly higher in sera from 81 patients with melanoma with American Joint Committee on Cancer (AJCC) stage I–IV disease, compared with 95 control sera from healthy individuals [78]. In another study antibodies against HERV-K *gag* and *env* transcripts have been observed in 16% (=51/312) sera of melanoma patients but not in 70 healthy controls [79]. Furthermore antibodies specific for a HERV-K trans-membrane envelope protein were reportedly found in 22% sera from patients with metastatic melanoma (N = 60), but again their prevalence in sera from 20 normal blood donors and patients with alopecia was nil [55]. There is evidence that the antibody response against HERV-K proteins in AJCC stages I–III melanoma patients is associated with poorer survival, and has thus been proposed as an additional prognostic factor [79].

However, the presentation of HERV-K epitopes on the surface of affected cells appeared also to represent the “*Achilles’ heel*” in the pathological changes induced by HERV-K [8]. These epitopes could indeed potentially serve as targets for immunity response aiming at repairing or eliminating the compromised cells.

Similarly to antiviral vaccines now used to prevent cervical cancer (anti-HPV vaccine) or hepatocellular carcinoma (anti-hepatitis B vaccine) preventive vaccines against usually non-expressed retroviral antigens may stimulate long lasting CD8+ T lymphocytic response in an otherwise vulnerable host that could then become able to eradicate early malignancies expressing these retroviral antigens [58].

Nearly 85% of malignant melanocytes express an antigen called HERV-K-MEL, a product of a pseudo-gene incorporated in the HERV-K *env* gene [25,80,81]. The HERV-K-MEL antigen, already previously defined as a *marker of melanoma risk*, is not present in normal tissues, but is significantly expressed in the majority of dysplastic and normal naevi, as well as other tumors like sarcoma, lymphoma, bladder, breast and ovarian cancer [25].

The *FEBrile Infections and Melanoma* (FEBIM) multicentre case–control study provided evidence how the Bacillus of Calmette Guerin (BCG) and vaccinia virus vaccination given in early childhood or acute infectious diseases acquired later in life were associated with a lesser melanoma risk [81]. This evidence was further examined and confirmed in another multi-centre case-control study conducted on 603 incident cases of malignant melanoma and 627 population controls (Table 1) [82].

**Table 1:** **Case-control study (FEBIM-1): Combined effect of infections and vaccinations on the risk of melanoma; Odds ratios (95% confidence interval) for melanoma risk, adjusted for study centre, gender, age, skin phenotype, freckling index, number of naevi and solar burns**[82]

	**Number of severe infections**	
	**0**	**≥1**
No vaccine	1.0	0.37 (0.10-1.42)
BCG or Vaccinia	0.57 (0.33-0-96)	0.29 (0.15-0.57)
BCG and Vaccinia	0.40 (0.23-0.68)	0.33 (0.17-0.65)

A protein bearing a high homology sequence of amino acids with the antigen HERV-K-MEL is expressed by BCG and vaccinia virus vaccine (Table 2). The yellow fever virus vaccine (YFV) was also found to express an antigen with a strict homology sequence of amino acids with HERV-K-MEL (Table 2) [38].

**Table 2:** **Comparison between amino acid sequence of HERV-K-MEL and proteins from different viruses**[38]


HERV-K-Mel	M	L	A	V	_	I	S	C	A	V
BCG	L	*	*	*	DV	V	P	I	*	*
Vaccinia virus	S	*	*	*	V	*	A	*	*	
Yellow fever virus	S	*	*	*	_	_	*	S	*	*

A = Alanine; L: Leucine; V = Valine; I = Isoleucine; S = Serine; M = Methionine; C = Cisteine; P=Proline; D=Aspartic Acid; G= Glycine; * = Identical amino acids; _ = Missing amino acid.

Sera from four Rhesus macaques before and four weeks after being administered with YFV were incubated with melanoma cells from two randomly selected patients: immune reactivity was observed at indirect immune-fluorescence in most apes post vaccination [Hunsmann & Krone 2005. Vaccination against malignant melanoma. European Patent EP1586330A1].

**Figure 2:**
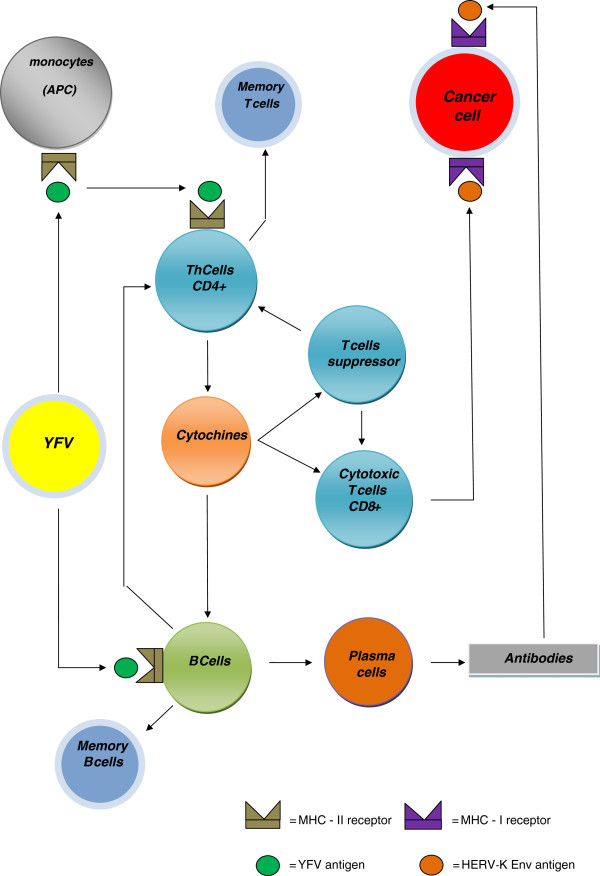
**Molecular mimicry and immunological response possibly triggered by the yellow fever virus vaccine (YFV), leading to cancer prevention.** APC= Antigen presenting cells.

This suggests that YFV might confer a protection against melanoma, by molecular mimicry (Figure 2).

To assess this protective effect, a cohort study (28,306 subjects vaccinated with YFV) and a case-control study nested in the cohort (37 melanoma cases vs. 151 tumours not expressing HERV-K-MEL) was recently performed in North-Eastern Italy [83]. The time elapsed since YFV up to end of follow up (TSV) was split into the following year intervals: 0-4; 5-9; 10+. In the case control study contrasting melanoma with tumors non-expressing HERV-K-MEL, the Odds Ratios (OR) for the above mentioned time bands adjusted for age and sex were 1.00, 0.96, (95% CI: 0.43-2.14) and 0.26 (95% CI: 0.07-0.96). The risk of melanoma was therefore reduced if YFV had been received at least 10 years before, as a result of prevention of tumor initiation rather than culling of already compromised melanoma cells [83].

Hodges Vasquez et al. [84] recently conducted a case-control study on 7,010 members of the US military to test the association between YFV and melanoma risk. Total cases of melanoma in this cohort were 638 diagnosed from 1999 to 2009 and each of them was contrasted with 10 healthy controls from active duty military service members. The study concluded that no significant association between YFV 17D and melanoma risk was found. However the maximum TSV was only 11.5 years and controls were presumably selected among healthy subjects. Selecting controls among individuals with malignancies other than melanoma from the same cohort of vaccinees (as done in the above Italian study) might influence the strength of the association, as study subjects would be a better choice. If the interaction between YFV and HERV-K-MEL prevents melanoma, healthy individuals could not be accepted as controls because some of them could be “cases of melanoma prevented by YFV” rather than simply subjects without disease. Prevention of melanoma could occur frequently because numerous infectious agents produce homologous epitopes capable of generating cross-reactive immunity.

**Figure 3:**
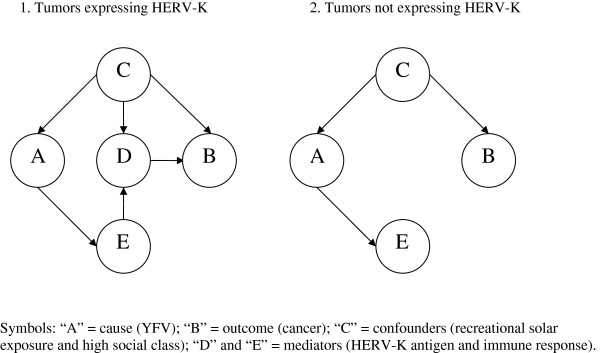
Directed acyclic graph (DAG) displaying the causal structure of the relationships between yellow fever vaccine (YFV), HERV-K, and cancer.

The presumed causal structure of the relationships between YFV, HERV-K, and melanoma can be conveyed in a directed acyclic graph (DAG) [85]. DAG #1 of Figure 3 relates to tumors expressing HERV-K-MEL. It can be seen that the cause (symbol A) is YFV; the confounders (B) include recreational solar exposure and high social class; the outcome (C) is cancer; and the mediators are expression of the HERV-K-MEL (D) and the immune response (E). The confounders may increase the use of YFV, affect expression of the HERV-K-MEL gene (and of other HERV-K genes) coding for putative oncogenic proteins, thus increasing the risk of cancer. YFV may induce a cross-reactive immune response that could decrease the expression of HERV-K genes and destroy or repair the cancer or its precursor cells by means of CD8^+^ T-lymphocytes. Since the corresponding paths are both open, YFV can be postulated to increase cancer through confounders (top path) as well to decrease it through immune response (bottom path). DAG #2 of Figure 3 relates to tumors not expressing HERV-K-MEL. It can be seen that YFV may be postulated to only increase cancer risk through confounders as the specific immune response is unlikely to affect these tumors.

### HERV-K and ovarian cancer

It was reported that multiple HERVs are simultaneously expressed in ovarian cancers [61]. Antibodies against HERV-K Env, HERV-E Env o ERV3 proteins have been detected in sera of patients affected by ovarian cancer, but not in healthy controls [61]. The presence of these antibodies provides indirect evidence of how HERV-K proteins might be immunogenic and act as tumor associated antigens.

The production of specific HERV-K antibodies indicates a lack of immunity tolerance and might signify that HERV-K expression during ontogenesis did not happen for ovarian cells, as proposed for melanoma [55]. Patients affected by ovarian cancer seem thus able to mount an immune response against specific HERVs, and immunotherapy against HERV-K proteins might be effective against ovarian cancer. In this regard it is important to note that HERV-K proteins are expressed in 90% of epithelial ovarian tumors, whereas their expression is nil in normal tissues or epithelial tissues from benign ovarian cancers [61].

Activation of HERV-K expression in ovarian cancer might happen in response to a transcriptional factor detected specifically in malignant epithelial cells of ovarian cancers [86-88]. This activation might be the result of hypo-methylation of HERV-K genomic DNA during tumor transformation and progression [61]. Retrotransposons have been reported as potential targets of hypo-methylation during cellular transformation [89]. An enhanced HERV-K expression has been reported as a result of DNA hypo-methylation in urothelial cancer [90] and germ line tumours [91]. A similar mechanism could occur also for ovarian cancer.

Assessment of HERV-K expression may therefore represent a new screening tool for ovarian cancer in the future, and served as target for detection, diagnosis and treatment of this neoplasm [61].

### HERV-K and breast cancer

Breast cancer is the leading cancer type and the second cause of cancer death among women of industrialized countries [92]. About 10% of breast cancer is attributable to genetic predisposition [93,94], with approximately 30% familial cases due to BRCA-1 or BRCA-2 genes mutations [95].

Earlier studies have suggested that protection from breast cancer is associated with early exposure to some common viruses, whereas exposure later in life increases the risk [96].

Breast cancer cell lines and tissues were found to express HERV-K env transcripts, whilst non-malignant breast tissues did not [93]. HERV-K expression was significantly higher in most breast cancer tissues than in normal breast tissues and a statistical correlation between estro-progestin stimuli and HERV-K *env* transcripts in breast cancer cells was reported by various authors [59,97,98]. In particular, HERV-K RT was found to be expressed in different human breast cancer cell lines but not in normal human breast tissues [98]. The exact role of HERV-K proteins in breast cancerogenesis is still obscure [98], but HERV-K *env* may contribute to cancer proliferation [57].

Expression of HERV-K *env* was recently detected in 66% (=148/223) human breast cancers inoculated into mice, and lymphnode metastatis were more likely to occur in HERV-K positive tumours [57]. Similarly to melanoma, HERV-K RT expression and humoral response against HERV-K antigens was identified as a novel marker and prognostic factor in disease free patients for breast cancer [57,79,98].

Monoclonal and single-chain antibodies against the HERV-K *Env* antigen proved capable of blocking proliferation of human breast cancer cells in vitro, inhibiting tumor growth in mice bearing xenograft tumors. In particular, immune-therapy selectively suppressed breast cancer cell growth but not non-malignant breast cells. Results showed that treatment of breast cancer cells with anti-HERV-K *Env* monoclonal antibodies induced apoptosis and activated the signaling pathway of TP53, a tumor suppressor protein with a key role in apoptosis and cell senescence [57].

## Summary

According to Hill’s criteria of modern epidemiology [99], an association is consistent when results are replicated in studies in different settings using various methods. This signifies that, for a relationship to be causal it has to be consistently found in different studies and different populations.

The above Italian study [83] raised the possibility that YFV is able to afford protection against melanoma at a very early stage of malignant transformation, perhaps preceding the clinical presentation of melanoma by many years. However, the evidence is based only upon three cases.

Further research appears recommended to confirm and elucidate the temporal pattern of the protection from melanoma attributable to YFV in other geographic areas and larger populations. It appears also appropriate to eliminate/attenuate the effect of potential confounders such as other vaccinations (namely BCG, vaccinia virus and possibly other vaccinations recommended for travelers to tropical areas) and especially socio-economic status, the latter being a significant risk factor for various malignancies, including melanoma [100,101] and breast cancer [100,102,103].

In view of the above, extending this investigation also to the potential protective effect of YFV on breast cancer appears indicated. Sarcoma, lymphoma, bladder and ovarian cancer should also be considered, as all these malignancies express significant levels of HERV-K *Env* epitopes [25,57-59,61].

If the above evidence were confirmed new possible pathways for the prevention of cancer could be opened.

Despite monoclonal antibodies against HERV-K Env proteins recently showing interesting results as a potential immunotherapeutic in breast cancer [57], cancer immunotherapy is still considered more complex and less advantageous than cancer immuno-prevention [80,104]. Furthermore, the efficacy of anti-HERV-K immunotherapy in the above study was only evaluated in mice bearing xenograft tumors, hence it should also be tested in breast cancer patients [57]. By contrast, YFV is largely affordable, reliable [105,106] and able to stimulate preventive cellular immunity against cancer, as antibody response is likely not to be the only immune mechanism involved against malignancies [38].

Several pathogens express antigens with an amino acid sequence homologous to the HERV-K-MEL epitope, but either the relevant proteins are not used to arrange the respective vaccines (e.g. tetanus toxoid and acellular pertussis vaccine), or most non-viable preparations are formulated to induce humoral response rather than cellular immunity [38]. Lastly, despite the evidence in favour of vaccinia and BCG vaccinations against the risk of melanoma [38], and the increasing global incidence of tuberculosis, the re-introduction of these two vaccines seems questionable [80].

## Abbreviations

BCG: Bacille Calmette-Guerin; CI: Confidence Interval; DNA: Deoxyribonucleic acid; FEBIM: Febrile Infections and Melanoma; HERV: Human Endogenous Retrovirus; HPV: Human Papilloma Virus; LTR: Long Terminal Repeats; OR: Odds Ratio; ORF: Open Reading Frames; RNA: Ribonucleic Acid; tRNA: transfer RNA; RT: Reverse Transcriptase; YFV: Yellow Fever Virus Vaccine; TSV: Time Since Vaccination.

## Competing interests

The authors declare that they have no competing interest.

## Authors’ contributions

LC and GM conceived the idea and drafted the paper; CS, GP, PV, EW contributed to the drafting of the paper. All authors read and approved the final manuscript.

## Funding

University of Padua.

## Pre-publication history

The pre-publication history for this paper can be accessed here:

http://www.biomedcentral.com/1471-2407/13/4/prepub
